# Accurate low and high grade glioma classification using free water eliminated diffusion tensor metrics and ensemble machine learning

**DOI:** 10.1038/s41598-024-70627-9

**Published:** 2024-08-27

**Authors:** Sreejith Vidyadharan, B. V. V. S. N. Prabhakar Rao, P. Yogeeswari, C. Kesavadas, Venkateswaran Rajagopalan

**Affiliations:** 1 Department of Electrical and Electronics Engineering, Birla Institute of Technology and Science Pilani, Hyderabad Campus, Hyderabad, 500078 India; 2 Department of Pharmacy, Birla Institute of Technology and Science Pilani, Hyderabad Campus, Hyderabad, 500078 India; 3 Department of Imaging Sciences and Interventional Radiology, Sree Chitra Tirunal Institute for Medical Sciences and Technology, Trivandrum, 695011 India

**Keywords:** Brain, MRI, Low-grade glioma, High-grade glioma, White matter, Diffusion tensor imaging, Two-compartment DTI model, Free water elimination, Machine learning, Ensemble learning, Cancer, Biomarkers, Neurology

## Abstract

Glioma, a predominant type of brain tumor, can be fatal. This necessitates an early diagnosis and effective treatment strategies. Current diagnosis is based on biopsy, prompting the need for non invasive neuroimaging alternatives. Diffusion tensor imaging (DTI) is a promising method for studying the pathophysiological impact of tumors on white matter (WM) tissue. Single-shell DTI studies in brain glioma patients have not accounted for free water (FW) contamination due to tumors. This study aimed to (a) assess the efficacy of a two-compartment DTI model that accounts for FW contamination and (b) identify DTI-based biomarkers to classify low-grade glioma (LGG) and high-grade glioma (HGG) patients. DTI data from 86 patients (LGG n = 39, HGG n = 47) were obtained using a routine clinical imaging protocol. DTI metrics of tumorous regions and normal-appearing white matter (NAWM) were evaluated. Advanced stacked-based ensemble learning was employed to classify LGG and HGG patients using both single- and two-compartment DTI model measures. The DTI metrics of the two-compartment model outperformed those of the standard single-compartment DTI model in terms of sensitivity, specificity, and area under the curve of receiver operating characteristic (AUC-ROC) score in classifying LGG and HGG patients. Four features (out of 16 features), namely fractional anisotropy (FA) of the edema and core region and FA and mean diffusivity of the NAWM region, showed superior performance (sensitivity = 92%, specificity = 90%, and AUC-ROC = 90%) in classifying LGG and HGG. This demonstrates that both tumorous and NAWM regions may be differentially affected in LGG and HGG patients. Our results demonstrate the significance of using a two-compartment DTI model that accounts for FW contamination by improving diagnostic accuracy. This improvement may eventually aid in planning treatment strategies for glioma patients.

## Introduction

Gliomas are the most prevalent type of brain tumors^1,2^. As per Global Cancer Statistics, 2020^3^, nearly 308,102 new cases of central nervous system cancer were diagnosed in 185 countries. Glioma is usually classified into low-grade glioma (LGG) and high-grade glioma (HGG)^2^. Based on the molecular characterization of central nervous system tumors, the survival time of HGG patients is shorter than that of LGG patients^1^. Therefore, it is important to diagnose brain tumors early to plan treatment strategies. Present clinical diagnosis is based on biopsy, which is an invasive procedure. This invasive procedure is expensive and prone to complications. Therefore, a non invasive quantitative approach using neuroimaging is essential to characterize the tumor. Magnetic resonance imaging (MRI) modalities T1-weighted, T2-weighted, fluid-attenuated inversion recovery (FLAIR), and T1-weighted contrast-enhanced (T1-c) images are currently employed in the qualitative radiological diagnosis of brain tumors. These conventional MRIs do not provide a quantitative assessment of the microstructural pathophysiological effects of gliomas on brain white matter (WM) tissue. Recent evidence^4–6^ shows that the pathophysiological process of the tumor is not restricted locally but also spreads globally through the WM pathways to other brain regions.

Diffusion tensor imaging (DTI) is a quantitative approach that uses water diffusion as a probe to assess brain WM tissue. Studies^7,8^ that measured fractional anisotropy (FA) and mean diffusivity (MD) values of the tumor region found that FA was able to classify LGG and HGG patients but not MD. In addition to FA and MD, others^9–12^ have used Westin’s indices, axial diffusivity (AD), and radial diffusivity (RD) to study different tumor regions (core, enhancing, and edema) and the normal-appearing WM (NAWM, meaning WM excluding the tumor regions) of the contralateral hemisphere. They found that the tumor affects the structural integrity of NAWM. All of the above studies used a single-compartment model for estimating the diffusion tensor. Aggressive tumor growth/invasion into the surrounding tissues (especially in HGG patients) leads to the accumulation of vasogenic edema^13–15^. This causes free water (FW) contamination in the WM tissue^16–18^. The FW in turn corrupts the FA and MD values^19,20^ and fiber tractography reconstruction^16,17^.

Therefore, in this study, (a) we will apply a bi-tensor model (two-compartment model, one for the brain tissue and one for FW separately), which is recommended when FW contamination is present^16–18^ because it improves the accuracy and specificity of the estimated DTI metrics. We hypothesize that the DTI metrics estimated using the bi-tensor model will classify LGG and HGG patients with high sensitivity and specificity values when compared to the single compartment model, (b) we will analyze DTI features of both the tumor and NAWM in LGG and HGG patients to identify neuroimaging non-invasive biomarkers, and c) we will use machine learning to classify glioma patients as opposed to the commonly used statistical inference methods, which are sometimes suboptimal^21^ because the relationship between predictor and dependent variables may vary for different datasets, which is unacceptable.

## Materials and methods

### Data acquisition

Among different types of brain tumor patients, we selected only the glioma patients who were categorized into LGG and HGG. An experienced radiologist (one of the authors) from Sree Chitra Tirunal Institute of Medical Science and Technology (SCTIMST), Thiruvananthapuram, India recruited the patients for this study. The glioma grading was determined after histopathological examination and biopsy. Retrospective imaging data of these glioma patients were collected from the SCTIMST database. The imaging was performed at the time of the biopsy. MRI scans of 86 brain tumor patients LGG (n = 39) and HGG (n = 47) were acquired. The patients MRI data included was acquired over the period spanning from March 8, 2013, to September 30, 2019. Since our clinical retrospective data was collected before the implementation of the WHO 2021 glioma grade classification criteria, the molecular diagnosis was not utilized for this cohort. Therefore, the classification relied solely on the histopathological criteria available at the time of data collection. The data for this study was collected as a part of the technological project in collaboration with SCTIMST. Data from this hospital were shared with us via a secured server keeping patient demographic details anonymous and sharing only the pathological details. Hence the patient demographic detail is unavailable in this study since we did not have direct access to this information.

### Imaging protocol

Since the data for this study came from a retrospective clinical repository, the patients were scanned either in a 1.5 T Siemens MRI scanner (Magnetom Avanto, Erlangen, Germany) or a 3 T General Electric Discovery MR750w scanner (Boston, United States). The MRI sequences include T1-weighted, T2-weighted, T1-c, and FLAIR, along with diffusion weighted images. Details about the imaging parameters are given in the Supplementary method section.

### Data processing

#### Tumor segmentation

**Fig. 1:**
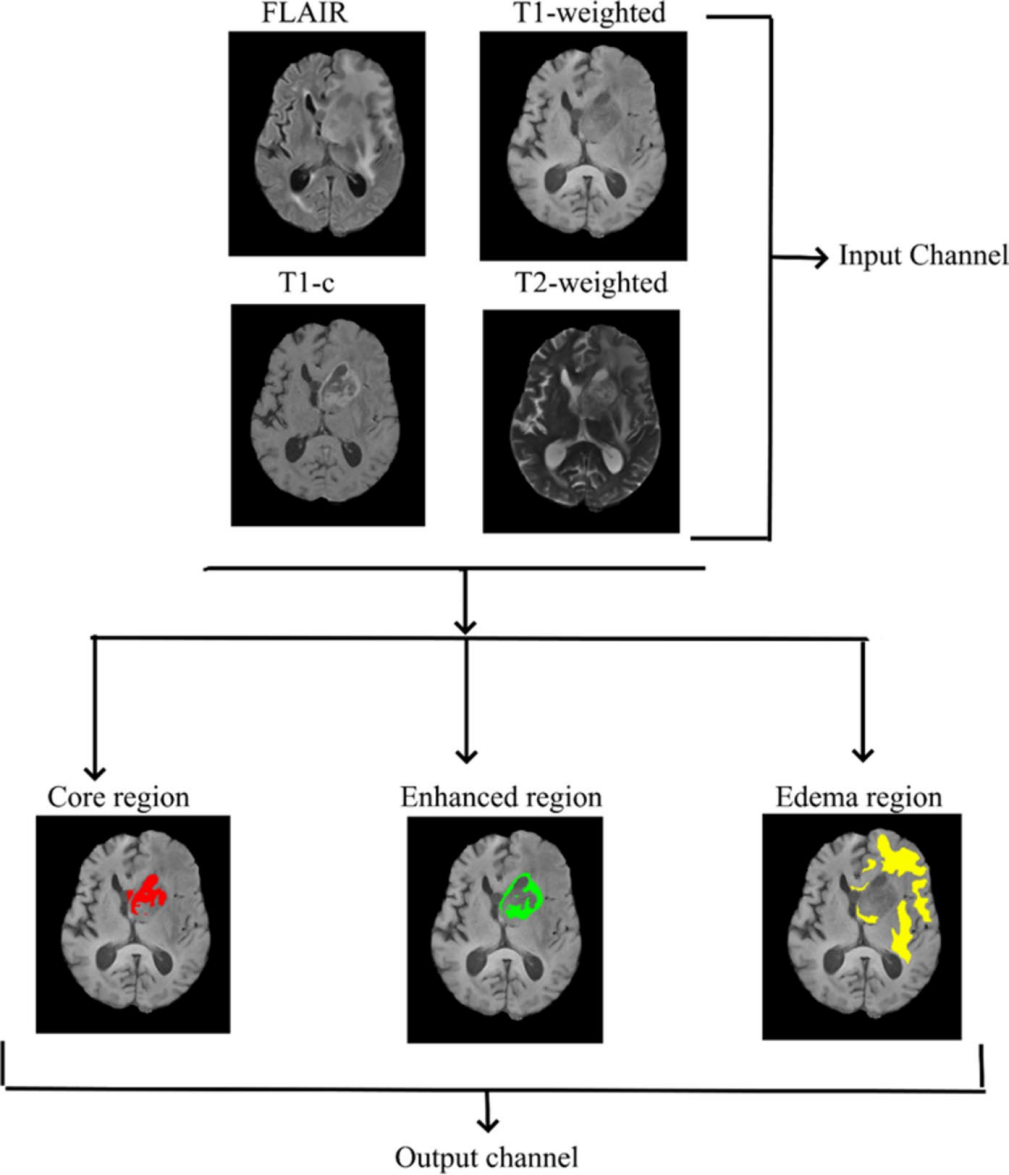
Tumor segmentation algorithm. Illustrates input and output images used in the nnU-Net tumor segmentation algorithm and segmented tumor regions given by the nnU-net algorithm.

The image pre-processing steps include skull stripping and bias field correction on T1-weighted, T2-weighted, FLAIR, and T1-c images. The FSL BET tool (version 6.0.4, https://fsl.fmrib.ox.ac.uk/fsl/fslwiki/FslInstallation) was used for skull stripping. Bias field correction was performed using the FSL FAST tool. Tumor segmentation for both LGG and HGG patients was performed using nnU-Net, a self-adapting automated deep learning framework^22^. Briefly, the nnU-Net architecture consists of four configurations, for more details refer to Isensee et al.^22^. nnU-Net takes four-channel inputs, namely T1-weighted, T2-weighted, FLAIR, and T1-c images, and outputs three segmented tumor images, namely core tumor, enhanced tumor, and edema, for each patient. For our dataset, the third configuration (3-D low resolution) of nnU-Net gave superior segmentation accuracy, but it failed to segment the resected tumor regions in a few of our patients. The missed resected tumor region was manually segmented and included. All the above steps were verified and validated by one of the authors who is an experienced radiologist. Figure 1 shows the segmented tumor regions in a typical brain tumor patient.

#### Diffusion tensor image processing

##### A. Standard DTI model (single-compartment model)

**Fig. 2:**
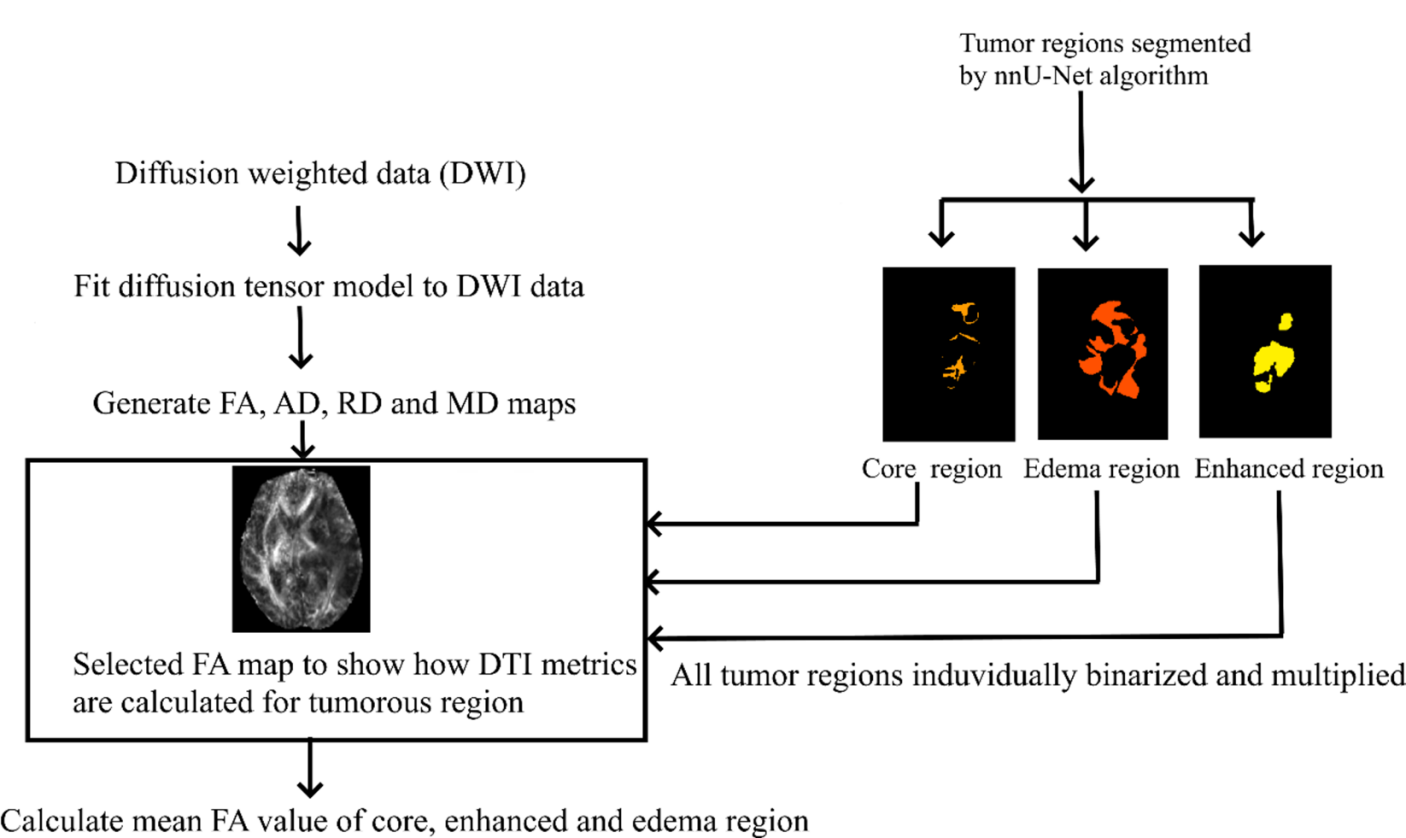
Feature extraction from tumorous regions**.** Illustrates how the mean FA value of the core, enhanced, and edema regions was measured for a typical patient. A similar procedure was followed to measure AD, RD, and MD of these tumorous regions.

Diffusion weighted images were processed using DSI-Studio (30 December 2020, build https://dsi-studio.labsolver.org/download.html). Affine registration was performed to correct for eddy current and motion distortion effects. The b-matrix was corrected for the gradient orientations. Brain extraction was performed on distortion-corrected images. A mono exponential fit using the linear least square optimization algorithm^23^ was used to estimate the diffusion tensor based on Eq. (1) given below:

1 $${\text{S}}_{{\text{j}}} = {\text{ S}}_{0} {\text{exp }}\left( { - {\text{b}}_{{\text{j}}} {\text{x}}_{{\text{j}}}^{{\text{T}}} {\text{D x}}_{{\text{j}}} } \right)$$

where S_j_ is the diffusion weighted image in the gradient direction x_j_ and a b-value of b_j_, S_0_ is the image with no diffusion weighting (b = 0), and D is the 3 × 3 diffusion tensor to be estimated. The DTI maps were then generated from the fitted diffusion tensor model. Average FA, AD, RD, and MD values for the core, enhanced, and edema regions for each patient were obtained by multiplying the binarized tumor segmented images with the DTI maps. Figure 2 shows the workflow details of DTI feature extraction from core, enhanced, and edema tumor regions.

##### B. Free water eliminated (FWE) DTI model (two-compartment model)

Single-shell diffusion weighted imaging is commonly employed in routine clinical imaging protocols like ours. We used the two-compartment model using Eq. (2) given below to fit the single-shell data as given by Golub et al.^24^ who used a regularized gradient descent algorithm for optimization:

2 $${\text{A }} = {\text{ f exp }}\left( { - {\text{b}}_{{\text{j}}} {\text{x}}_{{\text{j}}}^{{\text{T}}} {\text{D}}_{{\text{t}}} {\text{x}}_{{\text{j}}} } \right) \, + \, \left( {{1} - {\text{f}}} \right){\text{ exp }}\left( { - {\text{b}}_{{\text{j}}} {\text{D}}_{{\text{w}}} } \right),$$

where A = S_j_/S_0_, S_j_ is the image obtained after applying the gradient in the direction of x_j_ and b-value of b_j,_ S_0_ is the image with no diffusion weighting (b = 0), f is effective tissue-water fraction, (1 − f) is the effective FW fraction, D_t_ (diffusion tensor of tissue) and D_w_ (diffusion tensor for FW) are the bi-tensors to be estimated.

The bias-corrected diffusion weighted images (as processed for standard DTI model), binary brain mask, and corrected b-matrix files were used as input to the FW estimation code obtained from https://github.com/mvgolub/FW-DTI-Beltrami, with the pre-installed open source library DIPY^24^. After tensor fitting, FW eliminated FA, AD, RD, and MD maps were obtained. A FW map was also generated for each patient. The FW map characterizes water molecules that move freely and are not confined by their environment. Therefore, it represents the proportion of FW content present within each voxel. A visual illustration of the DTI maps obtained from both standard and FWE DTI models in this study is shown in Supplementary Fig. S1. Average FA, AD, RD, and MD values of the core, enhanced, and edema regions for each patient were obtained by multiplying the binarized tumor segmented images with the FA, AD, RD, and MD maps. The mean FW features of both tumorous and NAWM regions were discarded from this study to evaluate the performance of the standard and FWE DTI models using the same set of features.

##### C. Normal-appearing white matter

In the context of gliomas, NAWM is defined as the WM that is not visibly affected by the pathophysiological process of the tumor as it appears normal in conventional MRI sequences^25–31^. However, glioma studies have analyzed NAWM using different approaches. One of the approaches is to analyze a specific region of interest (ROI) in the WM surrounding the tumor region (core + enhanced + edema region) known as ipsilateral NAWM and in the WM region on the contralateral hemisphere where the tumor is absent known as contralateral NAWM. On the other hand, instead of analyzing a specific ROI in the contralateral/ipsilateral NAWM, other researchers^29–31^ have analyzed the entire WM region excluding the whole tumor region (i.e., core + enhanced + edema region), and termed it as NAWM because they found that the tumor infiltration process can diffuse beyond the vicinity of the tumor region thereby causing global microstructural changes to the WM tissue. They demonstrated that globally assessing WM excluding the whole tumor region can provide valuable information on overall WM integrity and health^29–31^. All the above-mentioned studies either used the term “contralateral NAWM/ipsilateral NAWM” when they focused on the region-specific analysis or “NAWM” when they aimed to globally evaluate the WM structural integrity due to the tumor. Similar to the previous studies^29–31^ we analyzed the entire WM region excluding the tumor and considered it as NAWM because the tumor can affect not only the neighboring/adjacent WM tissue (locally) but also can produce a diffuse effect in the other parts of the brain (globally). Also, more importantly, a significant percentage i.e., 42% of the patients (36 out of 86) considered in our study had tumor located in both the brain hemispheres hence, identifying contralateral and ipsilateral NAWM is not possible.

**Fig. 3:**
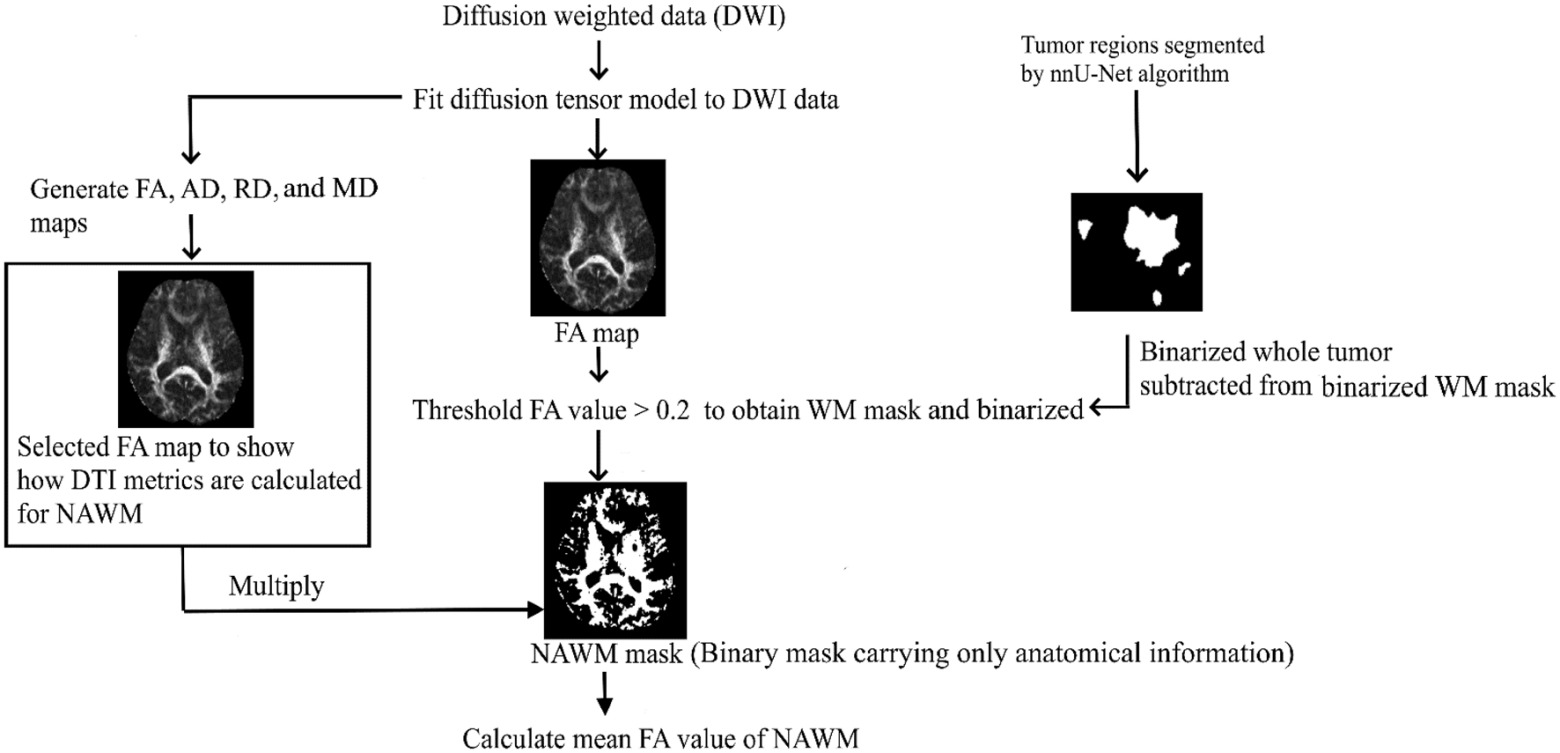
Feature extraction from the non-tumorous region. Illustrates how the mean FA value of the NAWM region was measured for a typical patient. A similar procedure was followed to measure AD, RD, and MD measures of the NAWM region.

To obtain NAWM images, the following processing steps were performed: (i) a WM mask image was extracted by thresholding the FA map (FA > 0.2) of each patient which is a standard method^25,32–34^, (ii) this WM mask image was subtracted from the segmented whole tumor region (which includes core, enhanced, and edema region) to obtain the NAWM mask. This NAWM mask was then multiplied with the FA, AD, RD, and MD maps. The whole brain average values for the above maps were obtained for each patient. We used the FSL (https://fsl.fmrib.ox.ac.uk/fsl/fslwiki) functions fslmaths and fslstats for the above calculations. The workflow diagram is shown in Fig. 3.

### Machine learning

**Fig. 4:**
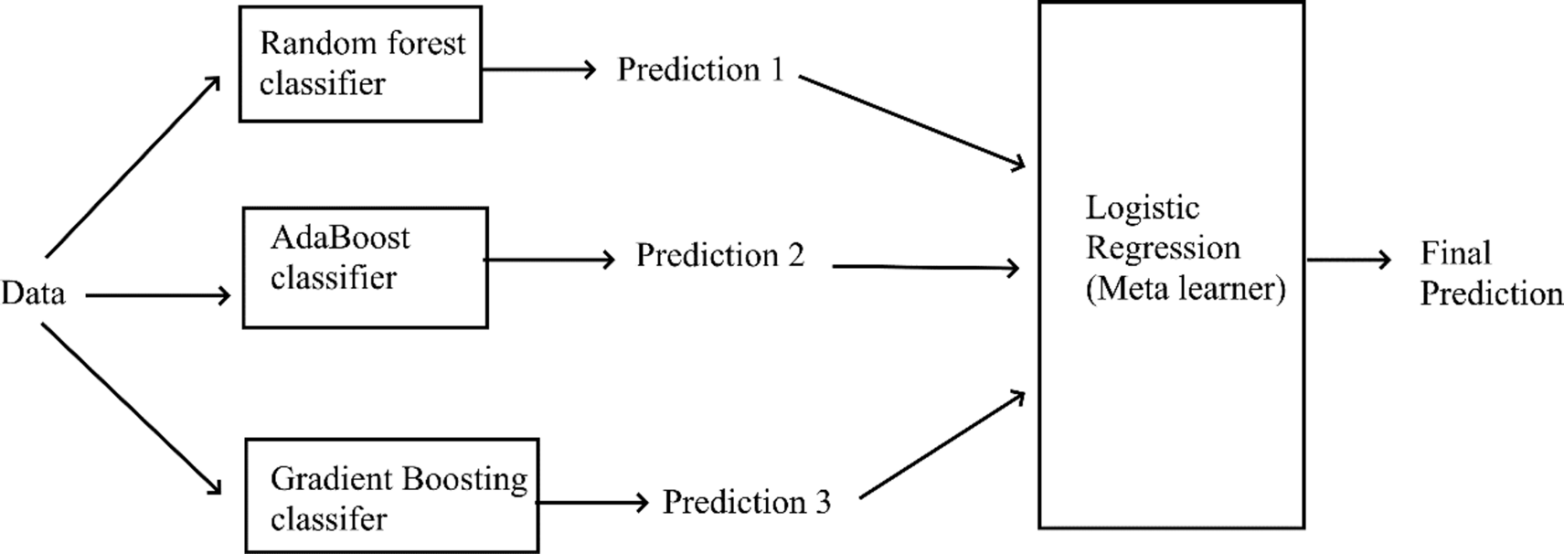
The EMLM architecture opted for this study. Predictions 1,2 and 3 from the individual single classifiers are provided to meta learner for obtaining the final output.

All the DTI features/measures obtained from the tumorous and NAWM using the standard DTI model and FWE DTI model (described above in "Diffusion tensor image processing") are given in Table 1. Different machine learning algorithms were used to classify LGG and HGG patients based on the above-mentioned features. These algorithms include support vector machine (linear and radial bias), random forest, naïve Bayes, AdaBoost, and gradient boost (these results are given in Supplementary Table S1). After assessing their performance (based on the sensitivity, specificity, and area under the curve of the receiver operating characteristics (AUC–ROC) score), we devised an advanced ensemble stacked learning-based architecture by combining the predictive power of individual models and providing each model’s prediction as an input to a meta learner. The ensemble machine learning model (EMLM) is shown in Fig. 4. Feature selection was performed using Weka software (version 3.8.6, https://www.cs.waikato.ac.nz/ml/weka/) using the CFS subset eval attribute selector and BestFirst search method. Features that were less correlated to each other and highly correlated with the target class (LGG-target value 0 and HGG target value 1) were selected. We adopted two approaches (1) all the features were used in the machine learning for classification and (2) only the Weka-selected features were used. We have presented only the performance metrics of the EMLM using Weka selected features in this article. The results obtained from the pre-selected features are provided in Supplementary Tables S2 and S3. Custom Python (version 3.9.7, Jupyter Notebook) codes were written for EMLM using the sklean, seaborn, NumPy, pandas, and matplotlib packages. The model parameters include n_estimators = 200 (for random forest, AdaBoost, and gradient boosting classifier), random state = 42, and train-test split = five-fold cross-validation.

**Table 1:** Shows the list of features extracted from the standard and the FWE DTI model in this study.

Region of interest	List of features extracted from both standard and FWE DTI model
Tumorous region	FA of the core region
	FA of the enhanced region
	FA of the edema region
	AD of the core region
	AD of the enhanced region
	AD of the edema region
	RD of the core region
	RD of the enhanced region
	RD of the edema region
	MD of the core region
	MD of the enhanced region
	MD of the edema region
Non-tumorous region (NAWM)	FA of the NAWM
	AD of the NAWM
	RD of the NAWM
	MD of the NAWM
Tumorous + non-tumorous region features	Combine all the features presented above in this column

### Ethical approval and consent to participate

The study was conducted according to the declaration of Helsinki. Institutional Ethics committee (IEC Regn No. ECR/189/Inst/KL/2013/RR-16) at Sree Chitra Tirunal Institute of Medical Science and Technology, Thiruvananthapuram, India approved this study waiving patient informed consent as this is a retrospective study. The approval number is IEC/1177. All procedures were performed in accordance with relevant guidelines.

## Results

### Machine learning results on DTI metrics derived from the standard DTI model

A total of 12 DTI features were measured from the tumorous region, including FA, AD, RD, and MD values of core, enhanced, and edema regions. For the NAWM region, four DTI features, namely FA, AD, RD, and MD, were measured. The above 12 features of the tumorous region + 4 NAWM region features were considered together as whole brain features. Weka attribute selection was performed to remove the redundant features for each of the ROIs mentioned in Table 1 and these selected features were used to train the EMLM. The results for EMLM evaluated on Weka selected features are shown in Table 3. Refer to Supplementary Table S2 for the results evaluated on pre-selected features. Note that the performance metrics reported in Table 2 are the average sensitivity, specificity, and AUC-ROC scores across all five folds.

**Table 2:** Shows the overall performance metrics of the EMLM when trained using the Weka selected DTI features of the tumorous region, NAWM region, and tumorous + NAWM region separately for the standard DTI model.

Region of interest	Weka attribute selection	Class	Sensitivity	Specificity	AUC-ROC score
Tumorous region	FA of the core and edema, AD of the enhanced	LGG	0.63	0.61	0.60
		HGG	0.66	0.61	0.65
NAWM	FA of the NAWM	LGG	0.70	0.61	0.66
		HGG	0.68	0.61	0.65
Tumorous + NAWM region	FA of the core and edema, AD of the enhanced FA of the NAWM	LGG	0.67	0.68	0.68
		HGG	0.64	0.64	0.64

### Machine learning results for DTI metrics derived from the FWE DTI model

A total of 12 DTI features were measured from the tumorous region, including FA, AD, RD, and MD values of core, enhanced, and edema regions. A total of four DTI features, i.e., FA, AD, RD, and MD were measured from the NAWM region. The above 12 tumorous region features + 4 NAWM region features were considered together as whole brain features. Weka attribute selection was performed to remove the redundant features for each of the ROIs mentioned in Table 1 and these selected features were used to train the EMLM. The results for EMLM evaluated on Weka selected features are shown in Table 3. Refer to Supplementary Table S3 for the results evaluated on pre-selected features.

**Table 3:** Shows the overall performance metrics of the EMLM when trained with the Weka selected DTI features of the tumorous region, NAWM region, and tumorous + NAWM region for the FWE DTI model.

Region of interest	Weka attribute selection	Class	Sensitivity	Specificity	AUC-ROC score
Tumorous region	FWE FA of the core and edema	LGG	0.86	0.84	0.84
		HGG	0.87	0.85	0.86
NAWM	FWE FA and MD of the NAWM	LGG	0.84	0.75	0.79
		HGG	0.80	0.79	0.78
Tumorous + NAWM region	FWE FA of the core and edema, FA and MD of the NAWM	LGG	0.92	0.90	0.90
		HGG	0.89	0.88	0.88

## Discussion

The important findings of this study are as follows: (a) DTI metrics derived from the FWE DTI model classified LGG and HGG with high sensitivity, specificity, and AUC-ROC score when compared to the standard DTI model, (b) we found that FW-corrected DTI is a robust approach to assess the WM structural integrity in glioma patients, and (c) when EMLM was trained with Weka selected features, i.e., FA of edema and core region, and FA and MD of NAWM region from the combined tumor + NAWM region features (16 pre-selected total features from FWE DTI model) classified LGG and HGG with superior performance metrics refer to Table 3. This demonstrates that (i) both tumorous and NAWM regions may be differentially affected between LGG and HGG patients, (ii) only 4 out of 16 features are required to classify LGG and HGG patients with good sensitivity, specificity, and AUC-ROC score indicating their potential role as neuroimaging biomarkers, and (d) EMLM gives better classification accuracy when compared with the routinely used single classifier model in brain tumor studies.

Comparison between Tables 2 and 3 shows that the DTI features derived from FWE DTI gave superior sensitivity, specificity, and AUC-ROC scores in classifying LGG and HGG patients when compared with the DTI metrics derived from the standard DTI model. We found that the values of FA, AD, and RD, which reflect WM integrity, were abnormally lower when using the standard DTI model as opposed to the FWE DTI model. For instance, when using the standard DTI model, the mean FA value for the core tumor region in LGG patients (n = 39) was 0.15 and that in HGG patients (n = 47) was 0.21. Similarly, for the enhanced region, the mean FA was 0.19 for LGG and 0.21 for HGG. On the other hand, when the FWE DTI model was used, these values were 0.44 in LGG, 0.50 in HGG for the tumor core region, 0.46 in LGG, and 0.47 in HGG for the enhanced region. The same phenomena were observed for the AD and RD measures. The reliability of the FWE DTI model is established by other researchers^17,24,35^. Similar to our study, other studies^17,24,35^ have also observed an increase in the FA values after correcting for FW contamination. Starck^35^ et al. also observed an increase in the FA value of tumorous regions after FW elimination. Golub et al.^24^ observed an increase in FA values in the artificially simulated lesion region after FW elimination. Pasternak et al.^17^ also found an increase in FA values in the region near the ventricles and edema after FW elimination, which eventually improved the accuracy of fiber tractography in those regions. The poor performance of the EMLM in classifying LGG and HGG patients when using the metrics from the standard DTI model can be attributed to FW contamination. This is evident from Tables 2 and 3. We can see that when using the FWE DTI model, an improvement in performance was observed in tumorous, NAWM, and tumorous + NAWM ROIs.

**Fig. 5:**
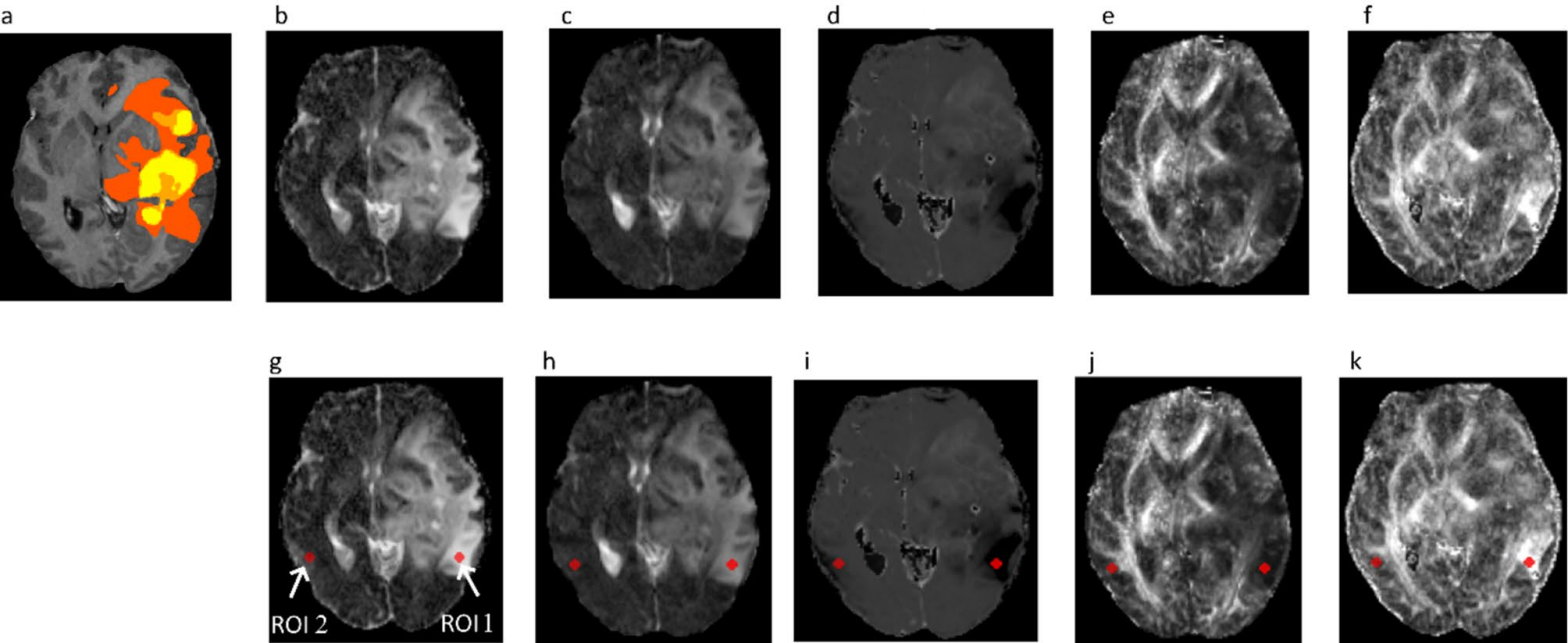
DTI analysis conducted on a typical HGG patient. (**a**) Shows the tumorous region superimposed on a T1-weighted image, (**b,g**) shows the FW map estimated using the FWE DTI model without and with ROIs, (**c**,**h**) shows MD map estimated using the standard DTI model without and with ROIs, (**d**,**i**) shows MD map estimated using FWE DTI model without and with ROIs, (**e**,**j**) shows the FA map estimated using the standard DTI model without and with ROIs, (**f**,**k**) shows the FA map estimated using FWE DTI model without and with ROIs. ROI 1 was placed in the edema region of the FW map, and ROI 2 was co-registered to the contralateral hemisphere using affine registration. All the above-mentioned operations were performed using FSL software.

To understand the effect of FW contamination, we performed an analysis by placing ROIs on the tumorous region and the corresponding region in the contralateral hemisphere (see Fig. 5). ROI 1 is located in the FW map at the high-water content region with mean FW value = 0.99 and ROI 2 is located in the contralateral hemisphere with a mean FW value = 0.68 (see Fig. 5b, g). From the previously mentioned figures and the mean FW values of ROIs 1 and 2, we can see that the contralateral hemisphere (ROI 2) is not contaminated with FW. We placed the same ROIs in the MD and FA maps of both the standard DTI (see Fig. 5c, h for MD map while Fig. 5e, j for FA map) and FWE DTI (see Fig. 5d, i for MD map while Fig. 5f, k for FA map) models. The MD values in ROI 1 and ROI 2 were 1.245 × 10^–3^ mm^2^/s and 0.894 × 10^–3^ mm^2^/s for the standard DTI model, whereas for the FWE DTI model, the mean MD values for ROI 1 = 0.685 × 10^–3^ mm^2^/s and ROI 2 = 0.873 × 10^–3^ mm^2^/s. Similarly, the FA values in ROI 1 = 0.145 and ROI 2 = 0.784 for the standard DTI model, and for the FWE DTI model, the mean FA values were ROI 1 = 0.59 and ROI 2 = 0.701. In comparison with healthy tissue (i.e., ROI 2) in the contralateral hemisphere, we can observe that (a) in ROI 1 (FW contaminated) of the standard DTI model, the FA and MD values were very low (0.145) and high (1.245 × 10^–3^ mm^2^/s), and (b) after FW elimination, i.e., using the FWE DTI model, the FA (0.59) and MD (0.685 × 10^–3^ mm^2^/s) measures returned to their optimal values. This analysis reveals that correction for FW contamination is required to obtain a robust estimate of the DTI measures.

The features of the FWE DTI model were able to classify LGG and HGG patients with superior sensitivity and specificity values when compared with the standard DTI model (see Tables 2 and 3). Now, if we look at Table 3 that shows the performance of features estimated from the FWE DTI model for the three different ROIs (i.e., tumorous region, NAWM region, and tumorous + NAWM region) we can infer that the tumorous + NAWM region gives superior classification accuracy when compared to the other two ROIs. This demonstrates that the features of both tumorous and NAWM regions play a critical role in classifying LGG and HGG patients. We believe that the tumorous and NAWM regions may be differentially affected in LGG and HGG patients. We attribute the subpar performance of the standard DTI model for the same ROI, i.e., tumorous + NAWM (Table 2) to the potential water contamination as reported in previous studies^17,24,35^. Our results show that the 4 features (FA of edema and core for tumorous region, FA and MD of NAWM) out of 16 features from the tumorous + NAWM region can provide superior classification accuracy between LGG and HGG when compared with using all 16 features. Because no other FWE DTI model-based brain tumor classification studies were available, it is not clear why the EMLM performed better on the above four features. However, similar to our observation, studies using the standard DTI model reported FA of the core tumor region^8,36^ as a valuable measure in classifying LGG and HGG. Similarly, other standard DTI model studies^37,38^ have reported that the tumor edema region can aid in glioma classification. In addition to the tumor region DTI measures, our analysis identified FA and MD of NAWM as important features in classifying LGG and HGG patients. Our results concur with those of other studies that have also reported FA of NAWM^39–41^ and MD of NAWM^25,29,31^ to be important parameters in glioma classification. 

Another important finding in this study is that the EMLM model performed better than individual classifiers (refer to Supplementary Table S1). Previous studies^42–46^ have employed various individual machine learning algorithms (single classifiers) to classify LGG and HGG based on radiomic features extracted from the tumorous region. However, none of these studies have specifically focused on training a machine learning model with features that capture the pathophysiological process of the tumor and its effects on WM structural integrity. Our findings align with those of other studies^47–49^ that reported good accuracy, sensitivity, and specificity when they employed ensemble models compared to individual classifiers in glioma classification. This is because ensemble learning leverages multiple machine learning classifiers to achieve improved predictive capability compared with single classifiers^50^. Another reason given by a study^51^ is that the diversity in data enhances the machine learning model’s discriminative ability. Our DTI features were diverse (both tumorous and NAWM regions), and this diverse data may have enabled the training process to capture discriminative information for robust classification. In addition, ensemble learning is more adaptable than single classifiers^52^. 

## Conclusion

Our results proved that the features derived using FWE DTI model was able to classify LGG and HGG patients with superior accuracy when compared to the standard DTI model. Our results also demonstrate that FW contamination correction is crucial for obtaining reliable DTI measures. Remarkably, the best classification results were achieved when utilizing only the FA of core and edema and the FA and MD of NAWM among 16 diverse DTI features (tumorous + NAWM region) extracted from the FWE DTI model. These measures can serve as non invasive neuroimaging biomarkers and can be confirmed in histopathological and longitudinal imaging studies. Furthermore, these findings suggest that assessing both the tumor and NAWM region can provide valuable insights into glioma classification. Our study also demonstrated that EMLM may be a more suitable machine learning approach than individual classifiers for features like ours.

### Supplementary Information

Supplementary Information.

## Data Availability

Due to the proprietary nature of the datasets obtained from medical institutions and in accordance with standard protocols governing patient data confidentiality, regrettably, we are unable to grant public access to the data. Requests for data access should be formally directed to venkateswaran@hyderabad.bits-pilani.ac.in.
