# Supplementary material for: Accurate low and high grade glioma classification using free water eliminated diffusion tensor metrics and ensemble machine learning
Source: Sci Rep. 2024 Aug 27;14:19844. doi: 10.1038/s41598-024-70627-9 (PMC11350135; doi:10.1038/s41598-024-70627-9)
Supplement: Supplementary file 1 — Supplementary Information. [file 41598_2024_70627_MOESM1_ESM.docx]

**Accurate low and high grade glioma classification using free water eliminated diffusion tensor metrics and ensemble machine learning**

Sreejith Vidyadharan^1^, BVVSN Prabhakar Rao^1^, P. Yogeeswari^2^ , C. Kesavadas^3^, Venkateswaran Rajagopalan^1^*

^1^Department of Electrical and Electronics Engineering, Birla Institute of Technology and Science Pilani, Hyderabad Campus, Hyderabad, 500078, India

^2^Department of Pharmacy, Birla Institute of Technology and Science Pilani, Hyderabad Campus, Hyderabad, 500078, India

^3^Department of Imaging Sciences and Interventional Radiology, Sree Chitra Tirunal Institute for Medical Sciences and Technology, Trivandrum, 695011, India

***Corresponding author**

Venkateswaran Rajagopalan

Department of Electrical and Electronics Engineering

Birla Institute of Technology and Science Pilani, Hyderabad Campus,

Hyderabad, 500078, India

Email: [venkateswaran@hyderabad.bits-pilani.ac.in](mailto:venkateswaran@hyderabad.bits-pilani.ac.in)

Phone: 04066303651

**Supplementary Information**

**Supplementary methods**

The MRI sequences acquired in the Siemens scanner have the following parameters. (1) T2-weighted images with a slice thickness = 5 mm, in plane resolution = 512 × 448, repetition time (TR) = 5860 ms, echo time (TE) = 110 ms, 2) FLAIR images were acquired with an in plane resolution = 512 × 448, slice thickness = 5 mm, TR = 9000 ms, inversion time (TI) = 2500 ms, TE = 89 ms, (3) T1-weighted images were acquired with slice thickness = 5 mm, in plane resolution = 320 × 270, TR = 468 ms, TE = 11 ms, (4) 3D gradient echo was used to acquire T1-c images whose imaging parameters include slice thickness = 0.9 mm, in plane resolution = 512 × 464, TR = 9 ms, TE = 3.34 ms and diffusion weighted images were obtained using single-shot echo planar imaging (SS-EPI) sequence along 20 or 30 diffusion weighted (b = 1000 s/mm2) directions (since the data are chosen from a routine clinical retrospective database) and one b = 0 s/mm2, in-plane resolution = 512 × 448, TR = 3500 ms, TE = 105 ms.

For the General Electric 3T scanner, the MRI sequences and the parameter settings are as follows: 1) T2-weighted images with a slice thickness = 5 mm, in plane resolution = 512 × 512, TR = 4863.08 ms, TE = 118.272 ms, 2) FLAIR images were acquired with an in plane resolution = 512 × 512, slice thickness = 5 mm, TR = 10000 ms, TI = 2570.35 ms, TE = 127.08 ms, (3) 2D T1-weighted images were acquired with slice thickness = 5 mm, in plane resolution = 512 × 512, TR= 2276.2 ms, TE = 21.408 ms, (4) 3D gradient echo was used to acquire T1-c images whose imaging parameters include slice thickness = 1.2 mm, in plane resolution = 512 × 512, TR = 9.272 ms, TE = 3.54 ms and diffusion weighted images were obtained using single-shot echo planar imaging (SS-EPI) sequence along 20 or 30 diffusion weighted (b = 1000 s/mm2) directions (since the data are chosen from a routine clinical retrospective database) and one b = 0 s/mm2, in-plane resolution = 256 × 256, TR = 8136 ms, TE = 73.7 ms.

**Supplementary Tables**

**Table S1**. Shows the results of individual classifiers used for this study.

| Machine learning algorithms | Tumorous region features | Normal appearing white matter (NAWM) features | Combined (tumorous + NAWM features) |
| --- | --- | --- | --- |
| Support vector machine (SVM): linear kernel | AUC-ROC score = 0.65 | AUC-ROC score = 0.64 | AUC-ROC score = 0.55 |
|  | Sensitivity = 0.70 | Sensitivity = 0.52 | Sensitivity = 0.40 |
|  | Specificity= 0.68 | Specificity= 0.69 | Specificity= 0.62 |
| Support vector machine (SVM): radial basis kernel | AUC-ROC score = 0.57 | AUC-ROC score = 0.46 | AUC-ROC score = 0.48 |
|  | Sensitivity = 0.60 | Sensitivity = 0.44 | Sensitivity = 0.54 |
|  | Specificity= 0.61 | Specificity= 0.47 | Specificity= 0.53 |
| Random forest | AUC-ROC score = 0.69 | AUC-ROC score = 0.59 | AUC-ROC score = 0.52 |
|  | Sensitivity = 0.70 | Sensitivity = 0.55 | Sensitivity = 0.47 |
|  | Specificity= 0.67 | Specificity= 0.54 | Specificity= 0.48 |
| Naïve Bayes | AUC-ROC score = 0.40 | AUC-ROC score = 0.45 | AUC-ROC score = 0.52 |
|  | Sensitivity = 0.45 | Sensitivity = 0.48 | Sensitivity = 0.54 |
|  | Specificity= 0.47 | Specificity= 0.47 | Specificity= 0.57 |
| AdaBoost | AUC-ROC score = 0.59 | AUC-ROC score = 0.64 | AUC-ROC score = 0.66 |
|  | Sensitivity = 0.61 | Sensitivity = 0.65 | Sensitivity = 0.68 |
|  | Specificity= 0.62 | Specificity= 0.68 | Specificity= 0.64 |
| Gradient Boost | AUC-ROC score = 0.60 | AUC-ROC score = 0.61 | AUC-ROC score = 0.55 |
|  | Sensitivity = 0.64 | Sensitivity = 0.49 | Sensitivity = 0.60 |
|  | Specificity= 0.55 | Specificity= 0.68 | Specificity= 0.58 |

**Table S2**. Shows the overall performance metrics of the EMLM when trained with the pre-selected DTI features of the tumorous region, NAWM region, and tumorous + NAWM region for the standard DTI model.

| Region of interest | Class | Sensitivity | Specificity | AUC-ROC score |
| --- | --- | --- | --- | --- |
| Tumorous  Region | LGG | 0.68 | 0.61 | 0.62 |
|  | HGG | 0.68 | 0.61 | 0.66 |
| NAWM | LGG | 0.72 | 0.48 | 0.56 |
|  | HGG | 0.62 | 0.48 | 0.60 |
| Tumorous + NAWM region | LGG | 0.62 | 0.70 | 0.68 |
|  | HGG | 0.56 | 0.69 | 0.62 |

**Table S3**. Shows the overall performance metrics of the EMLM when trained with the pre-selected DTI features of the tumorous region, NAWM region, and tumorous + NAWM region for the FWE DTI model.

| Region of interest | Class | Sensitivity | Specificity | AUC-ROC score |
| --- | --- | --- | --- | --- |
| Tumorous  Region | LGG | 0.76 | 0.79 | 0.78 |
|  | HGG | 0.81 | 0.79 | 0.78 |
| NAWM | LGG | 0.74 | 0.71 | 0.70 |
|  | HGG | 0.76 | 0.71 | 0.72 |
| Tumorous + NAWM region | LGG | 0.82 | 0.82 | 0.82 |
|  | HGG | 0.84 | 0.82 | 0.82 |

**Supplementary Figures**


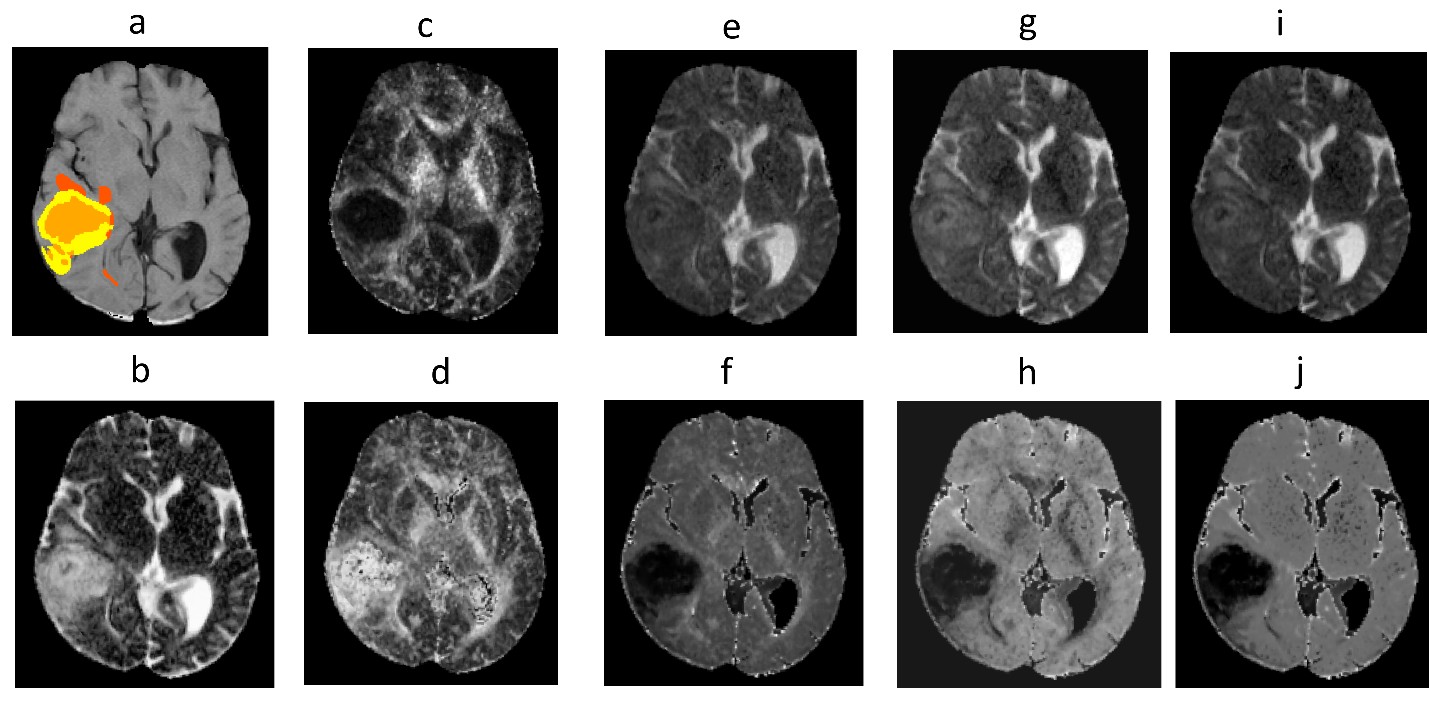


**Figure S1**. Shows a visual illustration of the DTI maps obtained from standard and FWE DTI models of a typical patient. (**a**) Tumorous region superimposed on a structural T1-weighted image for reference, (**b**) FW map obtained from the FWE DTI model, (**c**) FA map from standard DTI model, (**d**) FA map obtained from FWE DTI model, (**e**) AD map obtained from standard DTI model, (**f**) AD map obtained from FWE DTI model, (**g**) RD map obtained from standard DTI model, (**h**) RD map obtained from FWE DTI model, (**i**) MD map obtained from standard DTI model, (**j**) MD map obtained from FWE DTI model.
